# Preventing and treating childhood overweight and obesity in children up to 5 years old: A systematic review by intervention setting

**DOI:** 10.1111/mcn.13354

**Published:** 2022-03-25

**Authors:** Angela C. Flynn, Fatma Suleiman, Hazel Windsor‐Aubrey, Ingrid Wolfe, Majella O'Keeffe, Lucilla Poston, Kathryn V. Dalrymple

**Affiliations:** ^1^ Department of Women and Children's Health King's College London London UK; ^2^ Department of Nutritional Sciences, School of Life Course Sciences King's College London London UK; ^3^ Institute for Women and Children's Health King's Health Partners' London UK; ^4^ School of Food and Nutritional Sciences University College Cork Cork Ireland

**Keywords:** childcare setting, childhood obesity, diet, home, intervention, physical activity, school

## Abstract

The prevalence of childhood obesity is increasing worldwide with long‐term health consequences. Effective strategies to stem the rising childhood obesity rates are needed but systematic reviews of interventions have reported inconsistent effects. Evaluation of interventions could provide more practically relevant information when considered in the context of the setting in which the intervention was delivered. This systematic review has evaluated diet and physical activity interventions aimed at reducing obesity in children, from birth to 5 years old, by intervention setting. A systematic review of the literature, consistent with Preferred Reporting Items for Systematic Reviews and Meta‐Analyses (PRISMA) guidelines, was performed. Three electronic databases were searched from 2010 up to December 2020 for randomised controlled trials aiming to prevent or treat childhood obesity in children up to 5 years old. The studies were stratified according to the setting in which the intervention was conducted. Twenty‐eight studies were identified and included interventions in childcare/school (*n* = 11), home (*n* = 5), community (*n* = 5), hospital (*n* = 4), e‐health (*n* = 2) and mixed (*n* = 1) settings. Thirteen (46%) interventions led to improvements in childhood obesity measures, including body mass index *z*‐score and body fat percentage, 12 of which included both parental/family‐based interventions in conjunction with modifying the child's diet and physical activity behaviours. Home‐based interventions were identified as the most effective setting as four out of five studies reported significant changes in the child's weight outcomes. Interventions conducted in the home setting and those which included parents/families were effective in preventing childhood obesity. These findings should be considered when developing optimal strategies for the prevention of childhood obesity.

## Key messages

- The prevalence of childhood obesity is increasing worldwide, and effective public health strategies are needed to stem the rising trend of obesity rates.
- The aim of this systematic review was to evaluate diet and physical activity interventions aimed at reducing obesity in children, from birth to 5 years old, by intervention setting.
- The results of this systematic review show that interventions conducted in the home setting and those which included parents/families were effective in preventing childhood obesity.

## 1 INTRODUCTION

Over the last two decades, the global prevalence of overweight or obesity in children under the age of 5 has risen from 32 to 42 million (World Health Organisation, 2014). Once obesity is established in early life it may extend into adulthood, therefore creating a lifelong condition that is difficult to resolve (Geserick et al., 2018; Simmonds et al., 2016). Predictive modelling suggests, given the current levels of childhood obesity, that 60% of children today will have obesity by 35 years of age (Ward et al., 2017).

In the short term, children with obesity are at greater risk of adverse physical and psychological comorbidities (Pulgarón, 2013) as well as musculoskeletal difficulties, asthma and obstructive sleep apnoea (Narang & Mathew, 2012). In the longer term, there is an increased risk of morbidity, including type 2 diabetes, cardiovascular disease, cancer and premature death (Owen et al., 2009; Prospective Studies Collaboration, 2009). The substantial cost of treating the associated health implications has led to an intense focus on reducing rates of childhood obesity worldwide (Sonntag et al., 2016; World Health Organisation, 2016).

Among the potential causative factors, nutritional exposures in early life make a major contribution to the development of childhood obesity (Fogel et al., 2020), including the development of suboptimal eating habits and behaviours (Dalrymple et al., 2019), high intake of energy‐dense foods and a higher rate of food consumption (Fogel et al., 2017). Low levels of physical activity and the hours of TV viewing time are also implicated (Janssen et al., 2005). Longitudinal studies suggest that these dietary habits (Mikkilä et al., 2005; van Jaarsveld et al., 2014) and sedentary behaviours (Jago et al., 2005) established in early childhood may track into adult life (Rovio et al., 2018). Interventions that modify dietary intake and/or physical activity early in life, therefore, have the potential to improve lifelong health.

A recent Cochrane Review of childhood obesity interventions identified 153 randomised controlled trials (RCTs) targeting children from 0 to 18 years of age (Brown et al., 2019). Outcomes were stratified by age group and the authors reported that for children aged 0–5 years, dietary and physical activity interventions resulted in a modest reduction in childhood body mass index (BMI) and BMI *z*‐score (zBMI), and that the effect of interventions differed between settings. However, due to the scope and design of the Cochrane Review, it was not possible to identify which intervention was effective for whom and in which setting (Brown et al., 2019). Previous systematic reviews have summarised the evidence of intervention to reduce childhood obesity within a specific setting, such as home (Pamungkas & Chamroonsawasdi, 2019) or school/childcare (van de Kolk et al., 2019). Findings to date have been mixed, and the optimal setting to deliver intervention is not yet clear. As children spend a significant proportion of their day in either the home or school/childcare environment, interventions in these settings may potentially be more effective than those undertaken in a hospital/community‐based setting. To support obesity prevention strategies, it is important to evaluate interventions by setting to identify the optimal location of delivery. The aim of the present review was to systematically evaluate diet and physical activity interventions aimed at reducing obesity in children up to 5 years of age by intervention setting.

## 2 METHODS

This systematic review was conducted in accordance with the Preferred Reporting Items for Systematic Reviews and Meta‐Analyses (PRISMA) statement (Moher et al., 2009) (Supporting Information 1). The review was registered in the PROSPERO database, registration number CRD42020168311.

### 2.1 Inclusion and exclusion criteria

The inclusion and exclusion criteria were guided by the PICOS framework (participants, intervention, comparisons, outcomes and study design), summarised in Table 1. For inclusion, studies had to meet the following criteria: (1) RCTs that aimed to prevent or treat childhood obesity; (2) participants who were children aged up to 5 years old, family‐based interventions were also included as long as the age requirement for the child were met; (3) interventions with a defined dietary component or a mixed approach consisting of diet and physical activity components; (4) outcomes of childhood body composition including weight, height, BMI, zBMI, BMI‐percentile, percentage body fat and skinfold measures (SFM) and (5) studies published from the year 2010. This timeframe was selected to reflect up to date knowledge for overweight and obesity in early childhood. Studies meeting the following criteria were excluded: (1) nonrandomised and observational studies; (2) antenatal interventions that aimed to reduce the risk of childhood obesity or studies that aimed to assess breastfeeding initiation or duration, as these areas of research have recently been reviewed (Dalrymple et al., 2018; Rito et al., 2019); (3) conference abstracts and protocols; (4) interventions that did not mention the intervention setting; (5) absent or unpublished body composition data and (6) studies not reported in English.

**Table 1:** PICOS criteria

Parameter	Description
Population	Families/children aged up to 5 years old
Intervention	Diet only or diet and physical activity
Comparison	Control group
Outcome	Anthropometric measurements
Study design	Randomised controlled trials

### 2.2 Literature search

In January 2021, the following databases were searched from 1st January 2010 up to 31st December 2020: Medline, Embase and the Cochrane Central Register of Controlled Trials. The search strategies are outlined in Supporting Information 1.

### 2.3 Study selection and data extraction

Following removal of duplicates, screening of each title and abstract was independently completed by three reviewers (K. V. D., H. W.‐A. and F. S.) against the eligibility criteria. One hundred studies were assessed for eligibility and the full‐text articles were independently reviewed by the same three reviewers. For studies that met the inclusion criteria, data extraction was completed by two reviewers (H. W.‐A. and F. S.). A data extraction standardised template was developed, which included title, authors, date and location; intervention details: the numbers randomised to each trial arm, setting and a description of the intervention; primary and secondary reported outcomes and risk of bias. Any disagreement between the reviewers was resolved through a consensus opinion among the authors (K. V. D., H. W.‐A. and F. S.). The corresponding authors of two studies were contacted to confirm the study setting.

### 2.4 Data synthesis

A narrative synthesis was performed due to the heterogeneity of the study designs. Studies were categorised by intervention setting and included school/childcare, home, community, hospital/clinic, e‐health or mixed. School/childcare interventions were conducted within early years centres or school classrooms. Home‐based were defined as interventions that included a home visitation programme. Community‐based were delivered at community centres (including local churches and community health clinics). Hospital/clinic‐based interventions were delivered through primary care or outpatient clinics. E‐Health interventions were delivered via mobile phones, emails or videos and mixed interventions were those that included two or more of the aforementioned categories. Regardless of the setting, the interventions could be directed to parents and/or children through individual or group‐based sessions.

**Table 2:** Study characteristics

					Age at recruitment (range)	
Reference	Location	Design	Setting	Intervention type	Mean (SD) by group	Participants and baseline characteristics^a^
*School/childcare interventions*						
Bellows et al. (2013)	USA	RCT	Preschool	Diet and PA	3–5 years old I: 4.4 years (0.6) C: 4.3 years (0.6)	*N* = 263 I: *n* = 132 C: *n* = 131
Fitzgibbon et al. (2011) (postintervention), Kong et al. (2016) (1‐year follow‐up)	USA	RCT	Preschool	Diet and PA	4 years old I: 50.7 months C: 51.9 months	*N* = 618 children I: *n* = 325 BMI = 16.5 kg/m^2^ BMI percentiles: 5th–<85th *n* = 216 85th–<95th *n* = 62 >95th *n* = 45 C: *n* = 293 BMI = 16.6 kg/m^2^ BMI percentiles: 5th–<85th *n* = 189 85th–<95th *n* = 63 >95th *n* = 37
Hodgkinson et al. (2019)	UK	CRT	Sure Start Early Years' Centres	Diet, PA and Policy	2–4 years old I: 26.1 months C: 26.8 months	*N* = 81 parent‐infant dyads I: *n* = 47 parent–infant dyads Weight: 13.2kg C: *n* = 34 parent–infant dyads Weight: 13.2 kg
Kim et al. (2019)	South Korea	RCT	Daycare centres and kindergartens	Diet only	4 and 5 years old I: 4.5 years C: 4.4 years	*N* = 534 I: *n* = 254 BMI = 16 kg/m^2^ BMI percentiles: <10th *n* = 22 10th–<85th *n* = 269 >85th *n* = 33 C: *n* = 280 BMI = 16 kg/m^2^ BMI percentiles: <10th *n* = 19 10th–<85th *n* = 266 >85th *n* = 38
Lumeng et al. (2017)	USA	CRT	Classrooms	Diet and PA	4.1 ± 0.5 years NS + POPS: 4.10 (0.52) years NS + POPS + IYS: 4.12 (0.52) C: 4.12 (0.53) years	*N* = 697 I (NS + POPS): 224 BMI *z*‐score: 0.64 (1.18) I (NS + POPS + IYS): 255 BMI z‐score: 0.62 (1.18) C (HS): 218 BMI *z*‐score: 0.57 (1.18)
Natale et al. (2014)	USA	RCT	Childcare centres	Diet, PA and Policy	2–5 years old I: 3.5 years old C: 3.1 years old	*N* = 307 children I: *n* = 238 BMI percentiles: normal weight (<85th): *n* = 162 overweight (>85th to <95th): *n* = 39 Obesity (>95th): *n* = 32 C: *n* = 69 BMI percentiles: Normal weight (<85th): *n* = 46 Overweight (>85th to <95th): *n* = 10 Obesity (>95th): *n* = 11
Natale et al. (2017)	USA	RCT	Childcare centres	Diet, PA and Policy	3.9 years old I: 50.1 months C: 41.2 months	*N* = 1211 I: *n* = 754 BMI percentile: 65.13 C: *n* = 457 BMI percentile: 66.62
Salazar et al. (2014)	Chile	Pilot RCT	Daycare centres	Diet and PA	4.4 years old I: 4.4 years C: No data	*N* = 530 children recruited (a representative subsample of *n* = 265 was presented in the final analysis). I: *n* = 120 WFH *z*‐score: obesity 2.7, healthy weight 0.1 Percentage body fat: obesity 29.4%, healthy weight 21.7% C: *n* = 145 No baseline characteristics reported
Stookey et al. (2017)	USA	Pilot CRT	Childcare centres	Diet, PA and Policy	2–5 years old	*N* = 902 I: *n* = 522 C: *n* = 380
Verbestel et al. (2014)	Belgium	Pilot CRT	Daycare centres	Diet and PA	1.3 years old I: 15.8 months C: 14.9 months	N = 203 I: *n* = 126 BMI = 18 kg/m^2^ BMI z‐score: 1.29 Overweight: 22% C: *n* = 65 BMI = 17.4 kg/m^2^ BMI z‐score: 0.74 Overweight: 7.7%
Walton et al. (2015)	Canada	Pilot RCT	Early years centres	Diet and PA	2–5 years old I: 3.2 years C: 2.7 years	*N* = 48 parent‐infant dyads I: *n* = 27 parent–infant dyads BMI = 16.3 kg/m^2^ C: *n* = 21 parent–infant dyads BMI = 16.2 kg/m^2^
*Home*						
de la Haye et al. (2019)	USA	Pilot RCT	Home	Diet and PA	Birth (mean age at recruitment = 3.8 months)	*N* = 26 parent and child dyads I: *n* = 17 C: *n* = 9 WL *z*‐score for I + C = −0.46
Haines et al. (2018)	Canada	Pilot RCT	Home	Diet and PA	1.5–5 years old I: 3.3 years C: 3.1 years	*N* = 44 families I1 (4HV's): *n* = 17 Normal weight: *n* = 14 risk of overweight: *n* = 5 Mean age: 2.7 years I2 (2 HVs): *n* = 14 Normal weight: *n* = 13 risk of overweight: *n* = 4 C: *n* = 13 Normal weight: *n* = 8 risk of overweight: *n* = 8
Sherwood et al. (2015)	USA	Pilot RCT	Home	Diet and PA	2–4 years old I: 2.6 (0.72) years C: 2.9 (0.84) years	*N* = 60 parent‐child dyads I: *n* = 30 BMI percentile: 82.89 (8.48) BMI *z*‐score: 1.01 (0.36) C: *n* = 30 BMI percentile: 78.46 (12.20) BMI *z*‐score: 0.86 (0.43)
Tomayko et al. (2016)	USA	RCT	Home	Diet and PA	2–5 years old I: 4.0 (0.9) years C: 4.0 (0.9) years	*N* = 150 parent‐child dyads I: *n* = 67 BMI: 17.3 (1.6) kg/m^2^ BMI *z*‐score: 1.1 (1.0) BMI percentile: 78.8 (20.7) C: *n* = 83 BMI: 17.5 (2.5) kg/m^2^ BMI *z*‐score: 1.1 (1.2) BMI percentile: 75.5 (23).
Wall et al. (2019)	New Zealand and Australia	RCT	Home	Diet	12 months	*N* = 160 I: *n* = 80 BMI: 17.2 kg/m^2^ BMI *z*‐score: 0.4 C: *n* = 80 BMI: 17.6 kg/m^2^ BMI *z*‐score 0.7
*Community*						
Berry et al. (2011)	Mexico	RCT	Community centres and a local church	Diet and PA	2–5 years old	*N* = 56 mother‐child dyads I: *n* = 28 C: *n* = 28
Black et al. (2021)	USA	RCT	Community centres	Diet and PA	12 to 32 months I: 20.1 months C: 20.1 months	*N* = 277 toddler–mother dyads I: *n* = 186 BMI *z*‐score: 0.5 BMI percentiles: <85th *n* = 66 >85–<95th *n* = 9 >95th *n* = 15 C: *n* = 91 BMI *z*‐score: 0.63 BMI percentiles <85th *n* = 57 >85–<95th *n* = 18 >95th *n* = 15
Campbell et al. (2013) (mid‐ and postintervention), Hesketh et al. (2020) **(**2‐ and 3.5‐year follow‐up)	Australia	Cluster RCT	Community	Diet and PA	3.8 months I: 3.9 (1.6) months C: 3.9 (1.6) months	*N* = 542 parent and child dyads I: *n* = 271 BMI *z*‐score: −0.4 (1.1) C: *n* = 271 BMI *z*‐score: −0.5 (1.0)
Daniels et al. (2013)	Australia	RCT	Community Health Clinics	Diet	4.3 months I: 4.3 (1.0) months C: 4.3 (1.0) months	*N* = 698 mothers + infants I: *n* = 352 BMI: 16.46 (1.48) kg/m^2^ BMI *z*‐score: −0.36 (0.98) C: *n* = 346 BMI: 16.61 (1.48) kg/m^2^ BMI *z*‐score: −0.26 (0.98)
Skouteris et al. (2016)	Australia	RCT	Community centres	Diet and PA	2.7 years old I: 2.7 years C: 2.8 years	*N* = 171 I: *n* = 93 BMI *z*‐score: 0.66 BMI categories Normal weight: *n* = 56 At risk: *n* = 31 Overweight: *n* = 6 C: *n* = 78 BMI *z*‐score: 0.65 BMI categories Normal weight: *n* = 50 At risk: *n* = 25 Overweight: *n* = 2
*Hospital/clinic*						
Bocca et al. (2012)	Netherlands	RCT	Hospital out‐patient Clinic	Diet and PA	3–5 years I: 4.6 (0.8) years C: 4.7 (0.8) years	*N* = 75 children I: *n* = 40 BMI: 21.2 kg/m^2^ (2.9) BMI *z*‐score: 2.7 (1.0) C: *n* = 35 BMI: 21.0 kg/m^2^ (2.7) BMI *z*‐score: 2.7 (1.0)
Fisher et al. (2019)	USA	RCT	Clinic	Diet	3–5 years old I: 3.6 years C: 3.8 years	*N* = 119 mother and child dyads I: *n* = 59 BMI percentiles <85th *n* = 43 85–<95th *n* = 9 >95th *n* = 6 BMI: 16.5 kg/m^2^ BMI z‐score: 0.48 C: *n* = 60BMI percentiles <85th *n* = 38 85–<95th *n* = 13 >95th *n* = 7 BMI: 16.5 kg/m^2^ BMI *z*‐score: 0.68
Martínez‐Andrade et al. (2014)	Mexico	Pilot CRT	Primary care clinic	Diet and PA	3.4 years old I: 40.1 months C: 41.1 months	*N* = 306 I: *n* = 168 parent–infant dyads BMI: 17.3 kg/m^2^ BMI *z*‐score <1.0 (normal) n = 79 >1.0–<2.0 (at risk) *n* = 58 >2.0 (overweight) *n* = 31 C: *n* = 138 parent–infant dyads BMI: 17.3 kg/m^2^ BMI *Z*‐score <1.0 (normal) *n* = 56 >1.0–<2.0 (at risk) *n* = 61 >2.0 (overweight) *n* = 21
Quattrin et al. (2014)	USA	RCT	Primary care linic	Diet and PA	2–5 years old I: 4.6 (0.2) years C: 4.4 (0.2) years	*N* = 96 children I: *n* = 46 BMI: 20.4 (0.5) kg/m^2^ C: *n* = 50 BMI: 20.1 (0.4) kg/m^2^
*eHealth*						
Helle et al. (2019)	Norway	RCT	eHealth	Diet	3–5 months Mean age: 5.5 months	*N* = 718 parent‐child dyads I: *n* = 360 BMI: 17.04 kg/m^2^ BMI *z*‐score: −0.06 C: *n* = 358 BMI: 17.30 kg/m^2^ BMI *z*‐score: 0.11
Nyström et al. (2017,2018 **(**12‐month follow‐up)	Sweden	RCT	eHealth	Diet and PA	4.5 years old I: 4.5 (0.1) years C: 4.5 (0.1) years	*N* = 315 children Randomly assigned: I: *n* = 156, C: *n* = 138 Completed‐follow‐up I: *n* = 143 BMI: 15.9 (1.5) kg/m^2^ WFA *z*‐score: 0.00 (1.16) C: *n* = 138 BMI: 15.6 (1.2) kg/m^2^ WFA *z*‐score: −0.13 (1.04)
*Mixed*						
Stark et al. (2011)	USA	Pilot RCT	Mixed	Diet and PA	2–5 years old I: 4.4 (0.9) years C: 3.9 (1.1) years	*N* = 18 children I: *n* = 8 BMI percentile: 99% C: *n* = 10 BMI percentile: 97.7%

Abbreviations: BMI, body mass index; C, control; CRT, cluster‐randomised trials; HS, Head Start; HVs, home visits; I, intervention; IYS: Incredible Years Series; PA, physical activity; POPS, Preventing Obesity in Preschoolers Series; RCT, randomised controlled trials; WFA *z*‐score, weight‐for‐age *z*‐score; WFH *z*‐score, weight for height *z*‐score; WLZ, weight for length *z*‐score.

^a^ Data presented as mean or mean (SD).

### 2.5 Primary and secondary outcomes

The primary outcomes of this review were measures of childhood body composition, including BMI (z‐scores and percentiles), SKM, weight and height. The secondary outcomes included behavioural outcomes associated with eating habits and physical activity. Tables 3, 8 only report outcomes where a significant result was found. We deemed a study effective if at least one reported measure of obesity differed significantly between the intervention and control arms of the study.

**Table 3:** Content, delivery and outcomes in school/childcare‐based interventions

Reference	Study aims	Content	Delivery	Child obesity measures	Diet and PA outcomes
Bellows et al. (2013)	To test the efficacy of a Food Friends: Get Moving' With Mighty Moves programme. To determine whether children participating in the intervention improved their gross motor skill performance, physical activity levels and weight status	Diet and PA *Intervention*: 15–20 min/day of exercise, 4 days/week (72 lessons in total). 12‐week Food Friends nutrition programme, including Food Friends character to introduce children to new foods. Each class received an activity binder, music CD, activity mats, flashcards, puppets, scarves, balls, beanbags, ropes and parents' materials. Home connection materials were sent home throughout the programme, including educational handouts and a copy of the Mighty Moves music CD *Control*: 12‐week Food Friends nutrition programme *Duration*: 18‐week intervention	Classroom teachers to school children.	No intervention effect was found for weight status	No intervention effect was found for physical activity levels
Fitzgibbon et al. (2011) (postintervention) Kong et al. (2016) (1‐year follow‐up)	To assess whether a modified Hip‐Hop to Health Jr. intervention could be integrated into the everyday preschool curriculum and delivered by classroom teachers. The primary aim was to compare BMI and BMI *z‐* score changes between treatment groups	Diet and PA *Intervention*: Hip‐Hop to Health Jr. intervention consisted of interactive lessons each week with the aim of increasing PA and promoting healthy eating behaviours. Activities and themes included portion sizes, heart healthy exercises, Instead of TV, I could…, healthy snacks, colourful “Pyramid Puppets” representing food groups and cooking and food tasting sessions. Parental involvement included weekly newsletters that matched in‐class curriculum, Hip Hop to Health Physical Activity CDs used in the classroom to take home, and nutrition homework where if completed, parents would receive $5, for example, information on the calcium content of different types of milk and differences in fat content. *Control*: teacher‐delivered general health intervention control group. *Duration*: 14‐weeks	School teachers to children in a classroom twice a week for 20 min. Parents were provided with the option of attending twice weekly 30‐minute aerobic classes.	No significant outcomes	Greater level of moderate to vigorous PA (*p* = 0.03) in the intervention group postintervention. At 1‐year follow‐up, between groups a significant difference in HEI total score (*p* = 0.02). Significant differences in total fruit (*p* = 0.003), whole fruit (*p* = 0.02), SoFAAS (*p* = 0.02) and whole grains (*p* = 0.02).
Hodgkinson et al. (2019)	To prevent excess weight gain in preschool children within a childcare setting	Diet, PA and Policy *Intervention*: Training of early years classroom teachers to implement healthy eating and drinking policies, such as healthy meals/snacks served, food growing and active play. The *Healthy Heroes* curriculum pack from the educational health promotion resource *Be Active Eat Healthy* was used. Activities included the use of colourful characters doing healthy activities, for example, ‘going to the park’, puppets, songs and Change4Life materials *Control*: Usual activity *Duration*: 6 months	Early Years Centres staff during one to one and group sessions with parent–infants' dyads	Significant reduction in BMI *z*‐score: 0.49; 95% CI: 0.17–0.80, *p* = 0.002.	No significant outcomes in dietary behaviours. Changes in physical activity were not reported
Kim et al. (2019)	To assess the effectiveness of a NASA‐established nutrition‐themed Mission X: Train Like an Astronaut programme to improve the dietary behaviours and nutritional status of South Korean preschool children	Diet only *Intervention*: A nutrition‐themed curriculum intervention using an astronaut as a role model. Topics included a balance of food groups, high calcium foods, healthy versus unhealthy snacks and food. *Control*: usual activity *Duration*: 10 weeks	Weekly intervention delivered by a dietitian to school children and class teachers taught using the provided lesson plans and materials	No significant outcomes	A difference in total NQ score (*p* < 0.05) at 10‐week follow‐up, with a greater increase in the intervention group
Lumeng et al. (2017)	To determine the effect of an intervention to improve emotional and behavioural self‐regulation in combination with an obesity‐prevention programme on the prevalence of obesity and obesity‐related behaviours in preschoolers	Diet and PA *Intervention* POPS: based on social cognitive theory, POPS was developed to provide developmentally and culturally appropriate, evidence‐based and coordinated obesity‐prevention messages to preschoolers and their parents. Behavioural goals included increased frequency and variety of fruit and vegetable intake, reduced sugar‐sweetened beverage consumption, reduced screen time, cooking healthy meals at home, eating family meals, and eating healthy foods when eating out. *IYS*: programme that emphasises positive behavioural management techniques and enhances self‐regulation in young, low‐income children. It consisted of 60 lessons followed by smaller group activities that addressed self‐regulation skills, problem‐solving strategies and prosocial behaviour. The parent component consisted of lessons delivered by using video vignettes in 14 group sessions or 10 home visits that were reinforced with homework and follow‐up phone calls. Control: HS: education programme targeting evidence‐based obesity‐prevention behaviours embedded in Head Start *Duration*: 6–8 months	Both the parent and child components were delivered by a master's‐level nutrition educator (POPS) or master's‐level mental health specialist (IYS)	No effect on the prevalence of obesity	Sugar‐sweetened beverage intake (HS + POPS + IYS resulted in a greater decline than HS; *p* = 0.005).
Natale et al. (2014)	Aims included: (1) increase healthy eating habits and PA behaviours of 2–5‐year‐old children at the centre and at home, and (2) determine the feasibility and efficacy of the intervention in ethnically diverse child care centres to address health disparities	Diet, PA and Policy *Intervention*: Healthy Inside–Healthy Outside intervention involved introducing physical activity and snack policy, for example, creating school menu changes to reduce saturated fat content and having <60 min twice a week of screen time and >60 min of PA/day Homework and resources were sent to parents covering themes, such as how to introduce new foods, increase fruit and veg intake and PA, reduce screen time and modelling healthy eating behaviours.	Teacher component: Teachers and staff were trained on the role and rationale of the HI‐HO programme and were provided lessons to use with the children. Parent component: Monthly group parent educational dinners delivered by dietitians. A nutritionist worked with each child care centre to modify menus to make them compliant with the policies	No significant outcomes	No outcomes reported.
		*Control*: a visit from an injury prevention education mobile.			
		*Duration*: 6 months			
Natale et al., 2017	To evaluate HC2, a theoretically based, multifaceted obesity prevention intervention, targeting low‐income, multiethnic children. To assess whether the combination of two healthy role models; teachers and parents as nutritional gatekeepers would be more effective in maintaining BMI percentile and improving diet quality	Diet, PA and Policy *Intervention*: The Healthy Caregivers–Healthy Children intervention involved sessions in the first year delivered 6 monthly, and in Years 2 and 3, four booster sessions. The intervention involved environmental changes in line with policies outlined in the AAP Caring for Our Children, 3rd edition, for example, providing water as the main beverage, daily fruit and/or vegetables, physical activity >60 min per day, and screen time <30 min per week. These were used to design lesson plans for the school curriculum and during joint parent‐teacher group sessions, for example, education surrounding healthy food choices, snacks, budgeting, use of food stamps and food labels.	Sessions for children were delivered by trained teachers at the day care centre and the programme staff delivered the joint parent‐teacher group sessions	Significant effect on child BMI percentile: *β* = −1.95, SE = 0.97, *p* = 0.04	No significant outcomes in dietary behaviours. Differences in physical activity were not reported
		*Control*: an attention control injury prevention curriculum delivered by “Safety Sam,” a character who provided parents and teachers with home, car and child seat safety information			
		*Duration*: 2/3 years			
Salazar et al. (2014)	To assess the outcomes of a lifestyle intervention for 4–5‐year‐old children attending daycare centres, with the aim of increasing moderate to vigorous PA, reducing energy‐dense foods and body fat	Diet and PA *Intervention*: Education training delivered to teachers to implement in daycare centres based on Educative Guidelines in Nutrition and Physical Activity resources. Nutritionists and PA teachers provided weekly support. Families attended group sessions once to twice a month and were provided with educational leaflets. Topics included growth, nutrition and daily PA requirements for preschoolers, reducing energy‐dense foods, monitoring regular eating patterns and limiting screen time <1 h/day *Control*: usual activity *Duration*: not specified	Teachers received weekly training from nutritionists and PE teachers to deliver nutrition, PA and health promotion education to children and parents during “Healthy Days” group sessions	Significant difference for children with obesity and healthy weight in the IG compared with CG for: (1) fat‐mass index (obesity: −0.3 kg/m^2^ IG vs. +0.2 kg/m^2^ CG) (healthy: −0.1 kg/m^2^ IG vs. +0.1 kg/m^2^ CG *p* < 0.01) (2) Fat‐free mass index (obesity: +1.4 kg/m^2^ IG vs. +0. kg/m^2^ CG) (healthy: +1.0 kg/m^2^ IG vs. +0.5 kg/m^2^ CG *p* < 0.01) (3). Triceps and subscapular (obesity: −3.2 IG vs. +3.3 CG) (healthy: 1.0 IG vs. +2.0 CG *p* < 0.01) and (4). (4) Percentage body fat (obesity: −1.5% IG vs. +1.3% CG) (healthy: −0.7% IG vs. +1.0% CG *p* < 0.01)	Increase in time spent doing vigorous PA, a reduction in moderate PA, fat and energy intake (all *p* < 0.05). Behavioural changes were not compared between treatment groups.
Stookey et al. (2017)	To determine if the integration of HAP resources into routine public health nursing services significantly increased the number of nutrition and physical activity best practices adopted by childcare centres and improved changes in child obesity measures	Diet, PA and Policy *Intervention*: CCHP + HAP intervention involving healthy lifestyle promotion training and resources provided to childcare providers to implement the best nutrition and physical activity practices over one year; 16 h of voluntary training, which included menu planning, healthy lifestyle resources provided to parents and age‐appropriate physical activity. The intervention incorporated themes of self‐assessment and practice improvement for centres	CCHP public health nurses or health workers delivered the HAP resources to childcare centre staff in group sessions. Staff members from each childcare provider implemented changes within their centre to parent–infant dyads	Significant reduction in BMI percentile: mean (SE): −2.6 (0.9), *p* = 0.003 and BMI *z*‐score: mean (SE): −0.08 (0.03), *p* = 0.007	Behavioural outcomes were not reported
		*Control*: usual activity			
		*Duration*: 1 year			
Verbestel et al. (2014)	To evaluate the effects of a 1‐year family‐based healthy lifestyle intervention delivered through day‐care centres on children's BMI *z*‐scores and parental reported physical activity and diet‐related behaviours	Diet and PA *Intervention*: (1) practical tips relating to target health behaviour topics presented on a poster discussed every 2 months e.g. consumption of sweets, savoury snacks, fruit and vegetables, increasing PA and decreasing screen‐time, drinking milk and water, and (2) a tailored feedback form for parents to report their child's current health‐related behaviours and compare it with current recommendations. *Control*: control group *Duration*: 12‐months	Guidelines and tips were presented on a poster, and tailored feedback on activity and diet behaviours were developed by the researchers	At the 12‐month follow‐up, significant reduction in BMI *z*‐score in the intervention group (1.33–0.38): By time 0.93, *p* < 0.001 and time‐by‐condition –0.5, *p* < 0.05	No significant changes in health behaviours post‐ intervention. Significant effects by time were found in both groups: increases in soft drinks (*p* < 0.01), water (*p* < 0.01), sweet (*p* < 0.01) and savoury snacks (*p* < 0.01), and decreases in fruit (*p* < 0.01) and vegetable intake (*p* < .01) over 1 year. No differences in PA.
Walton et al. (2015)	To assess the effectiveness of a family‐based obesity prevention intervention that combined strategies to improve pre‐schoolers' nutrition and physical activity behaviours with an existing, empirically tested general parenting programme.	Diet and PA *Intervention*: Parents and Tots Together intervention involved weekly 2‐h group sessions. Activities included interactive children's programmes, learning ignore/distract strategies to reduce sugar‐sweetened drinks intake, group discussions and homework, for example, family physical activity, establishing routines, including sleep and identifying hunger/satiety cues	Weekly sessions delivered by trained group facilitators to both parents and infants in a group format	No significant outcomes	No significant outcomes.
		*Control*: Supervising for Home Safety sessions			
		*Duration*: 9 weeks			

Abbreviations: AAP, American Academy of Pediatrics; BMI, body mass index; CCHP, Child Care Health Program; CCHP+HAP, Child Care Health Program Plus Healthy Apple Program; CG, control group; HAP, Healthy Apple Program; HC2, Healthy Caregivers–Healthy Children; HEI, Healthy Eating Index; HS, Head Start; IG, intervention group; IYS, Incredible Years Series; NASA, The National Aeronautics and Space Administration; NQ, nutrition quotient; PA, physical activity; POPS, Preventing Obesity in Preschoolers Series; SoFAAS, calories from solid fat, alcohol and added sugar; SE, standard errors.

**Table 4:** Content, delivery and outcomes in home‐based interventions

Reference	Aims	Content	Delivery	Child obesity measures	Diet and PA outcomes
de la Haye et al. (2019)	To draw on social influence and social network theories to identify features of family social networks that support or hinder the outcomes of a novel early childhood obesity prevention programme delivered to mothers and infants to test the possibility that it may improve mothers' diet, physical activity and weights status as well as infant diet and weight trajectory	Diet, PA and behaviour *Intervention*: Home visitation programme plus obesity prevention: One home visit per week delivered in line with the family's preferences and cultural practices, which focused on dietary strategies, such as increasing fruit and vegetables and reducing foods high in saturated fat, SSBs and fruit juice and advised on healthy portion sizes. PA advice was as per PA guidelines for children and adults, focused on making play and PA a daily habit. *Control*: Home visitation programme only *Duration*: 6 months	Mothers and their infants enroled on the home visitation programmes with sessions run by home visitors	No significant outcomes	Decrease in SSBs in children whose mother's social network characteristic had contact daily/almost daily (*p* < 0.05). Significant decrease in SSB in children whose mother's network characteristics lived in the same neighbourhood and had contact daily/almost daily (*p* < 0.05)
Haines et al. (2018)	To test the feasibility and acceptability of the ‘Guelph Family Health Study' intervention, a home‐based obesity prevention intervention based on the healthy habits, happy homes intervention. The secondary aim was to examine the impact of the intervention on child dietary intake, activity level, sleep and adiposity	Diet and PA *Intervention*: Home visits for families focused on the diet (such as limiting SSB consumption and family meals), family PA, establishing sleep routines, increasing child sleep duration and limiting sedentary activities. Families had the option to set a behaviour change goal at each visit along with additional emails tailored to behaviour change goals, paper family routine trackers to record health behaviours and mailed support, which included strategies to support behaviour change, for example, indoor games to increase child PA. *Control*: monthly emails containing publicly available handouts on general child health *Duration*: 16 weeks	Two and four 1‐hour home visits delivered by health educators, all of whom were graduate students and registered dietitians every 4‐8 weeks.	Significantly lower fat mass percentage at 6 months for the two home visits intervention group. SD change = −4.96 (2.58), *b* = −3.54 (−6.11, −0.97), *p* = 0.01	Significant increase in fruit intake in the 4 HV and 2 HV intervention groups at 6 months (both *p* < 0.05).
Sherwood et al. (2015)	To evaluate the feasibility, acceptability and efficacy of a primary care‐based obesity prevention intervention, integrating paediatric care provider counselling and phone coaching to prevent unhealthy weight gain among preschool age children at risk of obesity or currently overweight.	Diet and PA *Intervention*: Parents received a one‐time counselling session during their ‘well child’ visit to raise awareness of their child's obesity risk and were provided with a pamphlet with information about obesity, injury prevention and their child's BMI percentile. The phone coaching session focused on healthy eating and PA (Busy Bodies, Better Bites). Each child received a ‘busy bag’, which contained resources, such as activity and dinner table conversation cards, dance music CD and inflatable beach ball). Calls focused on: (1) reducing screen time, (2) decreasing sweetened beverage availability, (3) increasing PA and (4) increasing availability of lower fat, lower calorie meals and snacks. *Control*: Healthy Tots/Safe Spots safety/injury prevention Contact Control Arm *Duration*: 6 months	One paediatrician visit and bi‐weekly over‐the‐phone contact with experienced interventionalists with bachelor's or Master's degrees in health behaviour, nutrition or exercise science	No difference in BMI *z*‐score or percentiles at the 6‐month follow‐up. Significantly greater reduction in BMI *z*‐score at 6 months for overweight children randomised to the ‘Busy bodies/better bites' intervention (*p* = 0.02) when time by treatment effect was moderated by child weight status	Significantly more minutes of moderate to vigorous physical activity per day for the intervention group at 6 months (*p* = 0.01).
Tomayko et al. (2016)	To test the efficacy of an obesity prevention toolkit, delivered using a community‐based participatory research approach either by home mentors or by monthly mailings to impact child and adult weight status, nutrition and PA behaviours and self‐efficacy for behaviour change at home	Diet and PA *Intervention*: Twelve sessions on a family‐based healthy lifestyle, which addressed one of the four target areas: eat more fruit and vegetables, consume less soda and added sugar, become more active and watch less TV. *Control*: mailed delivery toolkit *Duration*: 12 months	Twelve 60‐min home visits delivered bi‐monthly by community‐based trained home mentors who were tribal members with long‐standing employment in the community	No significant effect of toolkit delivery. Combined study arms showed significantly lower BMI: 17.4–17.9 kg/m^2^, SD = 2.2–3.0, *p* ≤ 0.01, BMI: *z*‐score: 1.1–1.2, SD = 1.1–1.0, *p* = 0.035 and BMI percentile: 76.8–80.1, SD = 22.0–19.6, *p* = 0.020 at 1 year postintervention	Significant increase in fruit and vegetable servings (*p* = 0.006) at 1 year in the combined study arms.
Wall et al. (2019)	To evaluate the effect of consuming growing up milk lite (GUMli) compared with standard cow's milk as part of a whole diet for 12 months, on body composition at 23 months of age	Diet only *Intervention*: Parents were instructed to provide 300 ml of growing up milk lite (GUMli) infant milk or the equivalent quantity of whole cow's milk (both in powder form and equal to 6 scoops) to their infants daily ***Control***: cow's milk *Duration*: 12 months.	NA	Significantly lower body fat percentage at 12 months for the intervention group: −2.19% (95% CI: −4.24, 0–0.15, *p* = 0.036).	Lower protein intake in the intervention group (*p* = 0.02) at 12 months

Abbreviations: FM, fat mass; NA, not applicable; PA, physical activity; SSB, sugar‐sweetened beverages.

**Table 5:** Content, delivery and outcomes in community‐based interventions

**Reference**	**Study aims**	**Content**	**Delivery**	**Child obesity measures**	**Diet and PA outcomes**
Berry et al. (2011)	A randomised pilot study to test the efficacy of the refined and adapted intervention in assisting Spanish‐speaking women to manage their weight and prevent type 2 diabetes and prevent excessive weight gain in their 2‐ to 4‐year‐old children	Diet and PA *Intervention*: Children: 45‐min of the Colour Me Healthy programme, and 1 h of free time on the playground. Colour Me Healthy is a programme that focuses on healthy nutrition choices and increased activity and using examples of new and colourful foods, games, songs and dancing *Control*: Wait‐list control *Duration*: 9 months	Community health educators delivered to women and their children at a local church and a community centre	Significantly lower BMI percentiles (*p: *‐0.03). Intervention: 86 (7.5) to 82 (6.8) Control 86 (11.1) to 88 (8.5)	Not reported
Black et al. (2021)	To evaluate whether maternal lifestyle or responsive parenting interventions would reduce the rate of BMI growth, increase PA, responsive mealtime interactions and diet quality in toddlers and mothers	Diet and PA *Intervention*: Two intervention groups: (1) responsive parenting intervention (Tot‐TOPS) focused on toddler diet, physical activity and behaviour, such as soothing without relying on food, tips to try new fruit and vegetables, appropriate portion sizes and recreational PA guide for toddlers, and (2) a maternal lifestyle intervention (Mom‐TOPS), focused on maternal diet and physical activity times, without referencing children's health behaviours, for example, healthy snack ideas, farmers market location with opening times. Outcomes for Mom‐TOPS group involved measures of both toddler–mother health behaviours. Both interventions involved five group sessions and three individual phone sessions. *Control*: The attention control intervention (Safe‐TOPS) provided a home safety intervention *Duration*: 4 months	Masters' level educators biweekly under the supervision of a psychologist to parents in group sessions. There were eight sessions in total (four group sessions, three individual telephone coaching sessions and a final group session) at the US Women, Infant and Children clinics	No significant outcomes	Increase in toddler total fruit intake at 6 months (*p* < 0.05). This decreased at 12 months but remained significant compared to controls. At 6 months, there were no effect of the interventions on PA. At 12 months, compared to the control arm, the MomTOPS group had an increase in PA of 24 min/day (CI 2.55, 46.32), with no intervention effect for the Tot‐TOPS group
Campbell et al. (2013) (mid‐ and post‐intervention) Hesketh et al. (2020) (2‐ and 3.5‐year follow‐up)	To test the effectiveness of an early childhood obesity prevention intervention delivered to first‐time parents in pre‐existing social groups would improve aspects of the child's diet, increase time spent physically active and reduce television viewing	Diet and PA *Intervention*: The INFANT trial used an anticipatory guidance framework to teach parenting skills to promote and build knowledge, skills and social support regarding infant feeding, physical activity and sedentary behaviours. Incorporated six key messages, for example, “Colour Every Meal with Fruit and Veg,” “Eat Together, Play Together” & “Off and Running” within a DVD and written materials with a newsletter sent out to reinforce messages. Parents who did not attend sessions were also sent intervention materials. *Control*: parents received six newsletters on nonobesity‐focused themes; all parents received usual care from child health nurses *Duration*: 15 months	First time parent groups at Maternal and Child Health Centre: Six 2‐h dietitian‐ delivered group sessions given quarterly	No significant outcomes	Significant decrease in noncore drinks at mid‐intervention for intervention group children (*p* = 0.03). 13.8% vs. 7.1%. Higher intake of fruit (0.23 g/day; CI 95% 0.01, 0.45), vegetables (0.28 g/day; 0.05, 0.51), vegetable variety (0.24 g/day; 0.03, 0.45) and water intake (0.41 g/day; 0.14, 0.67), and greater decrease in sweet snacks (− 0.24 g/day; −0.42, −0.07) in the intervention group at 2 years follow‐up
Daniels et al. (2013)	To evaluate an obesity prevention intervention that provided anticipatory guidance on early feeding to first‐time mothers	Diet only *Intervention:* Group sessions focused on early feeding targeted to the child's developmental stage and its association with positive child eating behaviour and weight status: (1) textures and tastes, (2) responsive feeding and (3) positive parenting. Intervention focused on healthy growth rather than obesity prevention. *Control*: Standard access to universal community child health services, which, at the mothers' initiative, could include child weighing and information via the Web or the telephone helpline *Duration*: 12 weeks	Six 1–1.5 h interactive sessions at community child health clinics delivered by a dietitian and psychologist bi‐weekly to mothers	No significant outcomes	Intervention mothers used nonresponsive feeding practices significantly less often and responsive feeding practices more often (*p* < 0.001)
Skouteris et al. (2016)	To evaluate the effects of the MEND programme on child dietary intake and eating habits, child physical activity/sedentary behaviours, zBMI and food neophobia	Diet and PA *Intervention*: MEND intervention is separated into 30 min of guided active play with parents, 15 min of healthy snack time with a role model ‘Max Moon' and 45 min of creative play for the children. Sessions focused on modifying dietary and physical activity behaviours and parenting behaviours. Examples of activities included how to overcome barriers to change, reading food labels, healthy meal/snack alternatives, causes of fussy eating portion sizes for adults and infants and ideas of how children can become involved in cooking and food/snack preparation. *Control*: waitlist control *Duration*: 10 weeks	Members of the community or community AHPs, such as health nurses and childcare workers, delivered the intervention to parents and infants within a group setting at community or maternal and child health centres	No significant outcomes	Postintervention, an increase in vegetables and less high‐energy snacks in the intervention group (both *p* < 0.05). The intervention group was more responsive to satiety cues (*p* = 0.047). These were not sustained at the 6‐ and 12‐month follow‐up

Abbreviations: AHPs, allied health professionals; BMI, body mass index; CI, confidence interval; *d*, standardised effect sizes; HEI, Healthy Eating Index; MEND, Mind, Exercise, Nutrition… Do It!; PA, physical activity; zBMI, BMI *z*‐score.

**Table 6:** Content, delivery and outcomes in hospital/clinic‐based interventions

Reference	Aim	Content	Delivery	Child obesity measures	Diet and PA outcomes
Bocca et al. (2012)	To evaluate the effect of a multidisciplinary intervention programme in overweight and obese children aged 3–5 years and their families when compared with usual care	Diet and PA *Intervention*: Group sessions provided dietary advice on normocaloric diets as well as education and advice to improve child eating behaviour. Physical activity advice focused on an active lifestyle and mimicked elementary school activity. Motor skills and having fun through exercise to improve wellbeing was emphasised. Behavioural therapy (just for parents) focused on enabling them to be healthy role models. *Control*: usual‐care programme *Duration*: 16 weeks	Dietary advice consisted of six 30‐min sessions delivered by a dietitian. Physical activity sessions consisted of 12 60‐min sessions delivered by a physiotherapist. Behavioural therapy for parents consisted of six 120‐min sessions delivered by a psychologist. Twenty‐five sessions in total (30 h in 16 weeks)	Significant decrease in BMI SD = 0.5 (0.3), 95% CI: 0.01–1.07, *p* = 0.05), BMI *z*‐score SD = 0.2 (0.1), 95% CI: 0.02– 0.42, *p* = 0.03) and WC‐z score SD = 0.3 (0.1), 95% CI: 0.04–0.60, *p* = 0.02) between the intervention and control group post‐intervention (16 weeks). Significant decrease in BMI SD = −1.0 (1.4), 95% CI −1.52 to −0.47, *p* = 0.03), BMI *z*‐score SD = −0.6 (0.5), 95% CI −0.82 to −0.44, *p* = 0.02), WC SD = 0.9 (4.6), 95% CI −0.73 to 2.59, *p* = 0.02) and WC *z*‐score SD = −0.4 (0.6), 95% CI −0.57 to −0.14, *p* = 0.01) for the intervention group at 12 months	Significant increase in fibre intake at 16 weeks in the intervention group (*p* = 0.02). 14.7 g/day vs. 13.1 g/day
Fisher et al. (2019)	To evaluate the efficacy of a ‘*Food, fun and families*’ parenting intervention for decreasing intake of SoFAS	Diet only *Intervention*: Group sessions on feeding practices, autonomy support and the family eating environment. Dietary advice cantered on reducing intakes of SoFAS such as SSBs *Control*: delayed treatment control *Duration*: 12 weeks	Twelve 60‐min sessions delivered by graduate‐level interventionists	No significant outcomes	Significant decrease (23%) in daily energy from SoFAS for intervention children vs. control postintervention (*p* = 0.01) and an increase in healthier beverages for children in the intervention group (*p* < 0.05)
Martínez‐Andrade et al. (2014)	To evaluate the effectiveness of an obesity‐specific prevention intervention in clinics culturally specific to Mexico to address childhood obesity in primary care settings	Diet and PA *Intervention*: 90 min of nutrition and PA education, and 30 min of interactive workshops, such as cooking, playing active games, creating shopping lists and healthy family meals *Control*: usual standard of care *Duration*: 6 weeks	2 h in 6 weekly group sessions, delivered by nurses and nutritionists to both parents and children. Home drop‐in visits and phone calls were used to encourage attendance at study visits	No significant outcomes	Significantly greater increase in vegetable intake at 3 months, and a reduction in sweet snacks and sugar added to drinks in the intervention group (all *p* < 0.05). This was not observed at 6 months
Quattrin et al. (2014)	To test the efficacy of treating 2–5‐year‐old overweight children with either a traditional approach focused on the child (control) or a behavioural intervention targeting the child and parent (intervention)	Diet and PA *Intervention*: delivered jointly to children and parents and focused on behaviour modification and parenting techniques, such as positive reinforcement, modelling healthy diet and activity and stimulus control. Parents received weight monitoring, dietary, sedentary and physical activity advice. Child weight loss goal was 0.5–1 lb/week *Control*: information control (IC) targeting weight control only in the child *Duration*: 12 months with 12‐month follow‐up.	Thirteen 60‐min sessions with practice enhancement assistants (who had a BSc/MSc in psychology, nutrition, exercise science or equivalent) or dietitians plus 10 phone calls during the intervention and three calls during the follow‐up.	Significant decrease in BMI percentile at 6 (I = 22.5, C = 26.9, *p* < 0.001), 12 (I = 23.8, C = 28.8, *p* = 0.005), 18 (I = 25.8, C = 30.9, *p* = 0.005) and 24 (I = 27, C = 33, *p* = 0.001) months in the intervention group versus control. Significant reduction in BMI *z*‐score at 6 (I = 1.69, C = 1.93, *p* < 0.001), 12 (I = 1.66, C = 1.90, *p* < 0.001), 18 (I = 1.66, C = 1.86, *p* < 0.01) and 24 (I = 1.61, C = 1.86, *p* < 0.007) months in the intervention group versus control	No significant outcomes

Abbreviations: BMI, body mass index; INFANT, Infant Feeding Activity and Nutrition trial; PA, physical activity; SoFAS, solid fat added sugar; SSB, sugar‐sweetened beverages; WC, waist circumference.

**Table 7:** Content, aims, delivery and outcomes in eHealth interventions

Reference	Content	Aims	Delivery	Child obesity measures	Diet and PA outcomes
Helle et al. (2019)	To evaluate whether anticipatory guidance on early feeding practices would lead to healthier child eating behaviours and food habits and more beneficial parental feeding practices. The secondary aim was to evaluate whether increased use of protective feeding practices would reduce the risk of later childhood obesity	Diet only *Intervention*: Videos focused on infant feeding topics, such as appropriate food types and textures, how taste preferences evolve and responsive feeding practices. Videos also included cooking films and recipes on how to make homemade baby food with widely available ingredients *Control*: routine care *Duration*: 7 months	Seven monthly emails with 3–5‐min‐long videos	No significant outcomes	The intervention group reported a significant increase in the times/day score for fruits and vegetables, more likely to eat the same dinner as the rest of the family or to be playing or watching TV during meals (all *p* < 0.004). Children were also more likely to eat breakfast and dinner (*p* < 0.035) with their family versus the control
Nyström et al. (2017, 2018) (1–year follow‐up)	To assess the effectiveness of a mobile health (mHealth) obesity prevention programme on body fat, dietary habits and PA in children aged 4.5 years	Diet and PA ‘MINISTOP’ intervention consisted of 12 different themes relating to diet, PA and sleep (healthy foods in general, breakfast, healthy small meals, physical activity and sedentary behaviour, candy and sweets, fruits and vegetables, drinks, eating between meals, fast food, sleep, foods outside the home and foods at special occasions. Each consisted of general information, advice and evidence‐based strategies on how to change unhealthy behaviours. *Control*: received a pamphlet on healthy eating and physical activity in preschool‐aged children based on the existing guidelines. *Duration*: 6 months	Delivered via a smartphone mobile application to the parents, who received regular push notifications but were also able to access the content at any time	No significant outcomes	Significant decrease in the intake of sweetened beverages for intervention group children at 6 months (*p* = 0.049)

Abbreviations: CG, control group; IG, intervention group; PA, physical activity.

**Table 8:** Content, aims, delivery and outcomes in mixed interventions

Reference	Aims	Content	Delivery	Child obesity measures	Diet and PA outcomes
Stark et al. (2011)	To assess the effectiveness of the intervention on the primary outcomes of reducing child BMI z‐score and parent weight and on secondary outcomes of child caloric intake and changes in the home food environment compared to control at 6 and 12 months	Diet and PA *Intervention*: Learning about Activity and Understanding Nutrition for Child Health; LAUNCH Two‐phase intervention: Phase 1 (intensive): Twelve weekly sessions (alternated between group and HVs). Group clinic sessions addressed dietary education, for example, snacks and beverages, breakfast/lunch and dinner and parents kept 7‐day food diaries with a goal intake of 1000–1200kcal/day, depending on the child's age. The physical activity component focused on decreasing screen time to <2 h/day and increasing PA to 60 min/day of active play. Children and parents were given pedometers with a daily step goal of 5000 and 10,000, respectively. Parents were taught child behaviour management skills to implement diet and activity changes. Phase 2 (maintenance): Twelve weeks of bi‐weekly group and home sessions focused on helping families to maintain changes in eating and activity by identifying barriers and problem‐solving. Parent group clinic sessions of 90 min (dietary education and PA) in Weeks 8, 10 and 12. *Control*: Enhanced standard care, presented the same diet and activity recommendations, but was delivered in a one‐time Paediatrician Counselling session. *Duration*: 6 months	Paediatricians and researchers in weekly and bi‐weekly sessions	Significant decrease in BMI *z*‐score for the intervention group versus control at 6 months, which was maintained at 12 months for each measurement: CI: −0.59 (0.17), −0.94 to −0.24, *d* = 1.7, *p* = 0.003, BMI percentile CI: −2.4 (1.0), −4.4 to −0.3, *d* = 1.2, *p* = 0.030, weight CI: −2.7 (0.8), −4.4 to −1.0, *d* = 1.7, *p* = 0.004	Significant decrease in energy intake at 6 and 12 months, high‐calorie beverages at 6 months, home high‐calorie foods at 6 and 12 months, increase in fruit and veg consumption at 6 months (all *p* < 0.05)

Abbreviations:  BMI, body mass index; HVs, home visits; PA, physical activity.

### 2.6 Study quality

The methodological quality of each study was assessed by two independent reviewers (H.W.‐A. and F.S.) using The Cochrane Handbook for Systematic Reviews of Interventions (Higgins & Green, 2011). The domains assessed included randomisation selection (selection bias), allocation concealment (selection bias) and follow‐up of participant dropout from recruitment to termination of the study (attrition bias). The studies were assessed using the Cochrane criteria for judging risk of bias and were ranked using high, low or unclear for each domain and overall risk of bias was assigned to each study. Inconsistent assessments were discussed, and a consensus was reached among the authors.

## 3 RESULTS

**Figure 1:**
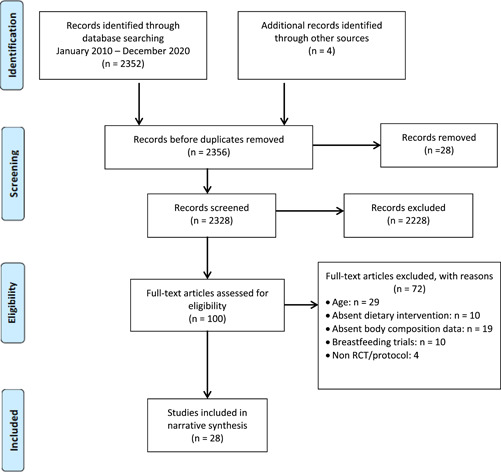
PRISMA flow diagram

The study selection process is presented in Figure 1. The literature search, including records identified through other sources, identified 2356 studies. Following the screening of titles and abstracts, 100 full‐text articles were assessed for eligibility. Twenty‐eight studies met the inclusion criteria. Reasons for exclusion included incorrect age of the child, antenatal studies, exclusively breastfeeding studies, incorrect study design, no dietary intervention and no childhood anthropometric data reported.

The studies were categorised by intervention setting; school/childcare (*n* = 11) (Bellows et al., 2013; Fitzgibbon et al., 2011; Hodgkinson et al., 2019; Kim et al., 2019; Lumeng et al., 2017; Natale et al., 2014, 2017; Salazar et al., 2014; Stookey et al., 2017; Verbestel et al., 2014; Walton et al., 2015), home (*n* = 5) (de la Haye et al., 2019; Haines et al., 2018; Sherwood et al., 2015; Tomayko et al., 2016; Wall et al., 2019), community (*n* = 5) (Berry et al., 2011; Black et al., 2021; Campbell et al., 2013; Daniels et al., 2013; Skouteris et al., 2016), hospital/clinic (*n* = 4) (Bocca et al., 2012; Fisher et al., 2019; Martínez‐Andrade et al., 2014; Quattrin et al., 2014), eHealth (*n* = 2) (Helle et al., 2019; Nyström et al., 2017) and mixed (*n* = 1) (Stark et al., 2011). The characteristics of the included studies are described in Table 2. The studies were conducted in the USA (Bellows et al., 2013; Black et al., 2021; de la Haye et al., 2019; Fisher et al., 2019; Fitzgibbon et al., 2011; Lumeng et al., 2017; Natale et al., 2014, 2017; Quattrin et al., 2014; Sherwood et al., 2015; Stark et al., 2011; Stookey et al., 2017; Tomayko et al., 2016), Canada (Haines et al., 2018; Walton et al., 2015), the UK (Hodgkinson et al., 2019), South Korea (Kim et al., 2019), Chile (Salazar et al., 2014), Belgium (Verbestel et al., 2014), New Zealand and/or Australia (Campbell et al., 2013; Daniels et al., 2013; Skouteris et al., 2016; Wall et al., 2019), Mexico (Berry et al., 2011; Martínez‐Andrade et al., 2014), the Netherlands (Bocca et al., 2012), Sweden (Nyström et al., 2017) and Norway (Helle et al., 2019). Six studies were pilot RCTs (de la Haye et al., 2019; Haines et al., 2018; Salazar et al., 2014; Sherwood et al., 2015; Stark et al., 2011; Walton et al., 2015), three were pilot cluster RCTs (Martínez‐Andrade et al., 2014; Stookey et al., 2017; Verbestel et al., 2014), three were cluster RCTs (Campbell et al., 2013; Hodgkinson et al., 2019; Lumeng et al., 2017) and 14 were RCTs (Bellows et al., 2013; Berry et al., 2011; Black et al., 2021; Bocca et al., 2012; Daniels et al., 2013; Fisher et al., 2019; Fitzgibbon et al., 2011; Helle et al., 2019; Kim et al., 2019; Natale et al., 2014, 2017; Nyström et al., 2017; Quattrin et al., 2014; Skouteris et al., 2016; Tomayko et al., 2016; Wall et al., 2019). Twenty‐three studies focused on changing dietary and physical activity behaviours (Bellows et al., 2013; Berry et al., 2011; Black et al., 2021; Bocca et al., 2012; Campbell et al., 2013; de la Haye et al., 2019; Fitzgibbon et al., 2011; Haines et al., 2018; Hodgkinson et al., 2019; Lumeng et al., 2017; Martínez‐Andrade et al., 2014; Natale et al., 2014, 2017; Nyström et al., 2017; Quattrin et al., 2014; Salazar et al., 2014; Sherwood et al., 2015; Skouteris et al., 2016; Stark et al., 2011; Stookey et al., 2017; Tomayko et al., 2016; Verbestel et al., 2014; Walton et al., 2015), four of which included policies to promote changes to the early years/childcare setting (Hodgkinson et al., 2019; Natale et al., 2014, 2017; Stookey et al., 2017). Five studies focused on modifying diet only (Daniels et al., 2013; Fisher et al., 2019; Helle et al., 2019; Kim et al., 2019; Wall et al., 2019).

The sample sizes across studies ranged from 18 (Stark et al., 2011) to 1211 (Natale et al., 2017) participants and age at baseline ranged from birth (Hodgkinson et al., 2019) to 5 years (Bocca et al., 2012).

### 3.1 School/childcare‐based interventions (*n* = 11)

One study used a diet‐only intervention, which was adopted from a nutrition‐focused US National Aeronautics and Space Administration (NASA) Program (Kim et al., 2019). The remaining 10 diet and physical activity interventions focused on reducing sugar‐sweetened beverages (SSBs), foods high in fat, sugar and salt, increasing consumption of water, milk, fruit, vegetable and new foods and promoting active play. Interventions included information on appropriate child portion sizes (Fitzgibbon et al., 2011), reducing screen time, examples of active play activities (Bellows et al., 2013; Lumeng et al., 2017; Natale et al., 2014, 2017; Salazar et al., 2014; Stookey et al., 2017; Verbestel et al., 2014), healthy snack and meal options (Fitzgibbon et al., 2011; Hodgkinson et al., 2019; Kim et al., 2019; Salazar et al., 2014; Stookey et al., 2017), high calcium foods (Kim et al., 2019) and a character‐based ‘Food Friends Program’ (Bellows et al., 2013). Four of the interventions implemented a health and wellbeing policy in the school or childcare setting, which aimed to influence a child's physical environment and sociocultural factors to create a setting that favours obesity prevention (Hodgkinson et al., 2019; Natale et al., 2014, 2017; Stookey et al., 2017). Dietary advice was provided by classroom/daycare centre teachers and staff (Bellows et al., 2013; Fitzgibbon et al., 2011; Hodgkinson et al., 2019; Natale et al., 2014, 2017; Salazar et al., 2014). Three of these studies included a structured training programme for the staff delivering the interventions (Bellows et al., 2013; Fitzgibbon et al., 2011; Hodgkinson et al., 2019). Interventions were also delivered by master's level educators (Lumeng et al., 2017), trained programme facilitators/staff (Stookey et al., 2017; Walton et al., 2015), dietitians and teachers (Kim et al., 2019; Natale et al., 2014) and through guidelines and tips presented on posters as well as tailored feedback on activity and diet‐related behaviours (Verbestel et al., 2014). The intensity of the interventions varied from twice weekly educational sessions (Fitzgibbon et al., 2011) to up to 6 months of educational sessions in the first year with four booster sessions delivered in Years 2 and 3 of the intervention (Natale et al., 2017). All interventions except for one (Verbestel et al., 2014) involved group‐based sessions for either children or parents or children and parents, with only one intervention also offering one‐to‐one sessions (Hodgkinson et al., 2019). Four interventions included family/parent group sessions (Lumeng et al., 2017; Natale et al., 2017; Salazar et al., 2014; Walton et al., 2015).

Five of the eleven studies reported a significant impact on childhood obesity measures with decreases in intervention group zBMI (Hodgkinson et al., 2019; Verbestel et al., 2014), BMI percentile (Natale et al., 2017; Stookey et al., 2017) and a significant change in fat mass, fat‐free mass, skinfold thicknesses and percentage body fat in the intervention group compared to controls (Salazar et al., 2014). Six of the eleven studies did not report a significant difference in obesity. However, four of these studies observed significant differences in dietary behaviours (Fitzgibbon et al., 2011; Kim et al., 2019; Lumeng et al., 2017; Salazar et al., 2014), of which two also reported a significant difference in physical activity (Fitzgibbon et al., 2011; Salazar et al., 2014) (Table 3).

### 3.2 Home‐based interventions (*n* = 5)

One dietary only intervention assigned participants to growing up infant milk, ‘GUMli’ (reduced protein with synbiotics and micronutrients) or whole, pasteurised cows' milk, both in powder form (Wall et al., 2019). The four combined interventions used a variety of strategies to modify dietary intake and physical activity behaviours, including incorporating culturally appropriate programmes (de la Haye et al., 2019; Tomayko et al., 2016), family‐based interventions to increase intake of fruit and vegetables (de la Haye et al., 2019; Tomayko et al., 2016), limiting consumption of foods high in saturated fat and SSBs (Haines et al., 2018) or SSBs and fruit juice while providing education on healthy portion sizes (de la Haye et al., 2019). In one study, families were given a paper family routine tracker to record their health behaviours and possible barriers to change (Haines et al., 2018). One intervention recruited children at risk of overweight or obesity (Sherwood et al., 2015). This intervention provided home counselling visits to raise awareness of obesity risk, obesity prevention tips, while a phone coaching programme for parents aimed to reduce screen time and increase physical activity (Sherwood et al., 2015). Interventions were delivered by trained home visitors who were matched to each family based on race, ethnicity and language preference (de la Haye et al., 2019), researchers (Wall et al., 2019), health educators (Haines et al., 2018), trained home mentors who were tribal members with long‐standing employment in the community (Tomayko et al., 2016) and paediatric care providers (Sherwood et al., 2015).

Four of the five studies reported a significant difference in the following measures of child obesity, including a reduction in percentage body fat at 12 months (Wall et al., 2019), a reduction in fat mass (Haines et al., 2018), BMI percentile and BMI *z*‐score (Sherwood et al., 2015; Tomayko et al., 2016). All five reported some significant differences in diet and physical activity behaviours, including a decrease in SSB intake (de la Haye et al., 2019), an increase in fruit and/or vegetable intake (Haines et al., 2018; Tomayko et al., 2016; Table 4).

### 3.3 Community‐based interventions (*n* = 5)

The community‐based interventions used a combination of dietary and physical activity focused interventions directed at individuals, rather than populations (Table 5; Berry et al., 2011; Black et al., 2021; Campbell et al., 2013; Daniels et al., 2013; Skouteris et al., 2016). One study used a family‐based behavioural model aimed at modifying dietary and physical activity behaviours through activities, such as creative play and skill development, to promote fruit and vegetable acceptance (Skouteris et al., 2016). Another consisted of two intervention groups: (1) responsive parenting intervention (Tot‐TOPS) focused on toddler diet, physical activity and behaviours, such as soothing without relying on food, appropriate portion sizes for toddlers and recreational physical activity guide for toddlers, and (2) a maternal lifestyle intervention (Mom‐TOPS), which focused on maternal diet and physical activity (Black et al., 2021). A further study used interactive group sessions informed by theoretical models (Daniels et al., 2013). Two interventions implemented family‐based programmes targeting healthy nutrition and activity, including Zumba classes and programmes that focused on healthy nutrition choices and increased activity (Berry et al., 2011; Campbell et al., 2013).

Dietary advice was provided by members of the community or community allied health professionals (e.g., maternal and child health nurse, childcare worker) (Skouteris et al., 2016), health educators (Berry et al., 2011; Black et al., 2021), dietitians (Campbell et al., 2013; Daniels et al., 2013) and psychologists (Daniels et al., 2013). The intensity of the interventions varied from quarterly sessions delivered during parents' regular group meetings (Campbell et al., 2013) to once a week for 3 months (Berry et al., 2011). The duration of the interventions varied from 10 weeks (Skouteris et al., 2016) to 15 months (Campbell et al., 2013). The community‐based interventions did not report a significant difference in obesity measures, except for one which reported a reduction in BMI percentiles (Berry et al., 2011). Four studies reported significant differences in dietary behaviours (Black et al., 2021; Campbell et al., 2013; Daniels et al., 2013; Skouteris et al., 2016). No studies reported an effect on offspring physical activity, except for one study (Black et al., 2021). The authors reported that there was no intervention effect at 6 months, at 12 months, compared to the control arm, the toddlers in the MomTOPS group had an increase in PA of 24 min/day (CI 2.55, 46.32), with no intervention effect for the toddlers in the Tot‐TOPS group.

### 3.4 Hospital/clinic‐based interventions (*n* = 4)

The hospital/clinic‐based interventions were conducted in primary care clinics (Martínez‐Andrade et al., 2014; Quattrin et al., 2014), a university‐based clinic (Fisher et al., 2019) or an outpatient clinic (Bocca et al., 2012). The only diet‐only study utilised a *food fun and families* parenting intervention to reduce children's intake of ‘empty calories’ from solid fat and added sugar (SoFAS) through group sessions for mothers with low‐income levels (Fisher et al., 2019). For the three diet and activity interventions, two recruited children with overweight and obesity (Bocca et al., 2012; Quattrin et al., 2014). These three studies aimed to modify dietary behaviour using a variety of approaches, including sessions with parents and children on improving diet, increasing physical activity and decreasing sedentary activity (Quattrin et al., 2014). One focused on eating breakfast while having an active lifestyle, consistent with elementary school exercises. Parents received behavioural therapy on how to become healthy role models for their children, changing family attitudes, removing unhealthy food triggers and knowing the difference between hunger and food cravings (Bocca et al., 2012). An obesity awareness and prevention curriculum was used by another study and included diet, healthy growth and physical activity workshops (Martínez‐Andrade et al., 2014). The duration of the interventions varied from 6 weeks (Martínez‐Andrade et al., 2014) to 12 months (Quattrin et al., 2014). For the diet‐only study, the intervention was provided by a graduate‐level interventionist (Fisher et al., 2019). The diet and physical activity interventions were delivered by dietitians (Bocca et al., 2012; Quattrin et al., 2014), nurses and nutritionists (Martínez‐Andrade et al., 2014) and practice enhancement assistants (Quattrin et al., 2014). The intensity of the interventions ranged from more than once per week (Bocca et al., 2012) to monthly (Quattrin et al., 2014). Three of the studies reported significant differences in dietary intake (Bocca et al., 2012; Fisher et al., 2019; Martínez‐Andrade et al., 2014) (Table 6), of which, two reported a significant reduction in body composition outcomes postintervention and at subsequent follow‐ups (Bocca et al., 2012; Quattrin et al., 2014).

### 3.5 eHealth‐based interventions (*n* = 2)

One study recruited families via social media and delivered guidance to parents in monthly video clips on feeding‐related topics, including the evolution of taste preferences in children, appropriate food types and textures and responsive feeding practices in addition to providing cooking and recipe information (Helle et al., 2019). The other aimed to change diet and physical activity behaviours via a freely accessible smartphone app for parents, which was compatible with both iOS (version 6.1.3 or higher) and Android (version 2.3.5 or higher) smartphones. The content was underpinned by social cognitive theory and behaviour change techniques and was based on guidelines for healthy eating and physical activity in preschool‐aged children (Nyström et al., 2017). The interventions were delivered by monthly video clips via email (Helle et al., 2019) or bi‐weekly via a smartphone application (Nyström et al., 2017). Neither study reported a significant difference in measures of childhood obesity. However, one study reported a significant improvement in dietary intake and eating behaviours (Helle et al., 2019; Table 7).

### 3.6 Mixed setting interventions (*n* = 1)

The mixed setting intervention was conducted in a clinic and at home (Stark et al., 2011) and recruited children with a BMI percentile ≥95. The dietary advice centred on meals, snacks and beverages and included a calorie target per day for each child. Parents were instructed to keep a 7‐day food diary and both parents and children were provided with pedometers and a daily step goal. Children also attended group sessions. Home sessions were delivered by psychology postdoctoral fellows while the group sessions were delivered by psychologists and research coordinators. The intervention was delivered in two phases: Phase 1 was delivered on a weekly basis for 12 weeks, alternating between group‐based clinic sessions and individual home visits and phase 2, which consisted of sessions every other week for 12 weeks, alternating between the clinic and home. The study reported a significant decrease in BMI percentile and *z*BMI at 6 and 12 months and an improvement in dietary intake (Stark et al., 2011; Table 8).

### 3.7 Quality of included studies

The overall quality of the included studies varied (Table S1). Nine studies were assessed to be at ‘high risk of bias’, 10 studies classified as ‘low risk of bias’ and the remaining 9 studies classified as ‘moderate’. Common sources of bias included no information on randomisation or high rates of attrition. Of the effective interventions, five were found to be at low risk of bias (Haines et al., 2018; Quattrin et al., 2014; Stark et al., 2011; Stookey et al., 2017; Wall et al., 2019), four were moderate (Bocca et al., 2012; Hodgkinson et al., 2019; Sherwood et al., 2015; Verbestel et al., 2014) and four were classified as high risk (Berry et al., 2011; Natale et al., 2017; Salazar et al., 2014; Tomayko et al., 2016).

## 4 DISCUSSION

This comprehensive and contemporary evaluation of interventions to prevent and reduce childhood obesity in young children has demonstrated differential effects of interventions on childhood obesity outcomes by intervention setting. Out of 13 studies that reported a significant difference in obesity measures, 5 were performed in a school/childcare setting, 4 in a home‐based setting, 2 in a clinic/hospital‐based setting, 1 in the community and 1 in a mixed intervention setting. The effective interventions all encompassed a diet and physical intervention, except for one, and they all recruited children over the age of 1 year. The duration of these studies ranged from 4 months to 2/3 years, with the majority of the successful trials intervening for at least 6–12 months. Five of these studies were found to be at low risk of bias, 4 were moderate and 4 were classified as high risk. No differences in obesity measures were reported in studies that utilised an eHealth intervention.

### 4.1 School/childcare‐based interventions

The school/childcare‐based interventions were diverse in their approach to reduce childhood obesity and included interventions aimed at the individual, family and policy levels. The interventions themselves were also delivered by a range of individuals from teachers to nutritionists. Five of the 11 interventions reported improvements in childhood obesity measures. The successful interventions included diet and physical activity components and family‐based approaches and the children's age at recruitment ranged from 1 to 5 years. Three of these also implemented policies to change the food environment in a childcare setting. Two previous systematic reviews have also reported that school or childcare interventions are effective at improving weight and body composition outcomes in 2–5‐year‐old children (Ling et al., 2016; van de Kolk et al., 2019). These reviews highlight that the most effective intervention strategies incorporate both family and the school/childcare environment. Our findings concur with this; we have also found that interventions including a policy‐based change to improve the food environment are effective at improving childhood outcomes (Hodgkinson et al., 2019; Natale et al., 2017; Stookey et al., 2017).

### 4.2 Home‐based interventions

Four home‐based interventions improved childhood obesity measures (Haines et al., 2018; Sherwood et al., 2015; Tomayko et al., 2016; Wall et al., 2019), a finding consistent with previous reviews (Brown et al., 2019; Narzisi & Simons, 2020). Three of these studies included family/parent involvement (Haines et al., 2018; Sherwood et al., 2015; Tomayko et al., 2016). Parental involvement has been shown to be necessary when implementing dietary changes in young children (Alderman & Headey, 2017). Parents have control over the dietary intake of their child, particularly during the early years; therefore, interventions that target the family as a whole may have a more positive impact on encouraging the development of healthy eating patterns in offspring (Savage et al., 2007). Our finding is consistent with a systematic review and meta‐analysis that assessed the influence of parental practices on child promotive and preventive food consumption behaviours and found that alongside food availability, parental modelling showed a strong association with both healthy and unhealthy food consumption (Yee et al., 2017).

In the present review, three of the four studies that reported a significant difference in childhood body composition outcomes also reported improvements in dietary behaviour, of which two studies reported positive changes in physical activity. Previous research examining the role parents have in shaping their children's physical activity and sedentary behaviours has suggested that parental involvement may increase daily physical activity and contribute to the prevention of weight gain (Pyper et al., 2016). One of the interventions utilised a different dietary strategy to reduce childhood obesity, evaluating standard cow's milk against lower protein milk for 12 months in 1‐year‐old children in New Zealand. Percentage body fat was modestly lower in the children assigned to the lower protein milk arm (Wall et al., 2019), which aligns with previous studies reporting an association between high protein intake in infancy and a higher BMI in later childhood (Günther et al., 2007; Michaelsen & Greer, 2014). Larger RCTs are required to examine whether this approach is an effective intervention to reduce childhood obesity.

### 4.3 Community‐based interventions

The community‐based interventions were directed at individuals, rather than populations. One of the five interventions conducted in a community setting reported an improvement in childhood obesity measures. Several studies reported greater dropout rates from parents of lower socioeconomic status, with one noting that dropout/missed sessions were due to the cost of travel, childcare and work commitments (Skouteris et al., 2016). This may have influenced the level of engagement and outcomes of the intervention, with possible barriers of travelling to a community setting if it is not a frequent destination, in comparison to interventions set within participants' own home or a school/childcare setting.

### 4.4 Clinic/hospital‐based interventions

Our review found that two of the four clinic/hospital‐based interventions led to differences in childhood obesity measures between treatment groups (Bocca et al., 2012; Quattrin et al., 2014). Both studies recruited either children with overweight or obesity and utilised an intense dietary and physical activity intervention, which led to some improvements in dietary intake. Interventions set in this environment targeted both children and parents; however, this setting may be less amenable to addressing other social and physical environment influences on behaviour, which may limit effectiveness, compared to other settings.

### 4.5 eHealth

Two studies in this review evaluated the use of parent‐focused eHealth interventions in reducing obesity in young children, neither of which reported a difference in childhood obesity measures. Some eHealth interventions have been shown to be successful in adults (Hutchesson et al., 2015); however, there is a paucity of data in children. Hammersley et al. (2016) assessed parent‐focused eHealth interventions in children and adolescents and out of eight included studies, no significant differences in obesity measures were reported. The use of eHealth interventions may provide the opportunity to overcome barriers that arise from in‐person sessions, such as providing greater scheduling flexibility and reaching at‐risk families (Jacobs et al., 2016; Reinwand et al., 2015). More research is needed to establish whether parent‐focused eHealth interventions could play a role in preventing obesity in young children.

### 4.6 Future implications

This systematic review has identified that interventions conducted in the home setting and those which included parents/families are the most effective in reducing obesity in children from birth to 5 years. School/childcare interventions which included policy‐based interventions were also effective. Furthermore, studies that solely recruited children with overweight or obesity all reported significant differences in the measure of BMI between the trial arms (Bocca et al., 2012; Quattrin et al., 2014; Stark et al., 2011). This suggests that interventions in children with overweight or obesity have the potential to have a greater influence on body composition outcomes. Future reviews of childhood obesity interventions could therefore stratify outcomes by BMI category to explore this observation further. We know that a child's food choices and lifestyle decisions are controlled by parents/caregivers at home; this may also extend to childcare settings where 76% of UK children under 5 years old attend a form of part‐time or full‐time childcare (Scaglioni et al., 2018). With evidence that one‐third of a child's daily calories are consumed within schools; interventions that target both parents and policies within the childcare/school settings should enable continuity in modelling healthy food patterns, therefore preventing mixed messages and compromising learnt behaviours (Liu et al., 2019), while optimising opportunities to promote better health outcomes.

### 4.7 Strengths and limitations

Strengths of this systematic review include the use of a structured process and framework guided by the Centre for Reviews and Dissemination (2009) and PRISMA statement. We used a comprehensive search strategy to identify relevant studies. However, this review is limited by the exclusion of non‐English‐reported studies, increasing the risk of selection bias. Comparison across interventions was challenging due to the wide variation in follow‐up time points and the variety of childhood obesity measures used. The classification of intervention settings may have introduced bias by creating a focus on one or two specific intervention settings that have been shown in this review to be successful, with the risk of discounting the potential effectiveness of other settings. An alternative approach would have been to assess interventions by population level (policy) or individual level. Finally, this review included several pilot studies, which may not have been adequately powered to detect differences in child obesity measures.

## 5 CONCLUSION

This contemporary and comprehensive review highlights the differential effect of interventions on measures of child obesity by setting. Interventions conducted in a home setting and those that included parent/family involvement more frequently led to improvements in childhood obesity measures than those in the community or hospital settings or involving eHealth coaching. We also found that studies that recruited children with overweight or obesity were also effective. The successful interventions used a variety of strategies to modify diet and physical activity behaviours and the implementation of policy to influence the early‐years environment appeared to be effective. The success of interventions may be attributed to the involvement of adults who are key to the child's care, for example, the entire family/parents and/or teachers, known to play an important role in obesity prevention and treatment in this age group. In particular, in childcare settings children are often influenced heavily by their peers or in the case of the home environment, by siblings/family members. These findings should be considered when developing optimal strategies for the prevention or treatment of childhood obesity.

## AUTHOR CONTRIBUTIONS

The research question and design study were formulated by Kathryn V. Dalrymple. Titles, abstracts and full‐text articles were independently screened by Kathryn V. Dalrymple, Hazel Windsor‐Aubrey and Fatma Suleiman. Data extraction and analysis were carried out by Hazel Windsor‐Aubrey and Fatma Suleiman. The writing of the article was completed by Kathryn V. Dalrymple, Hazel Windsor‐Aubrey, Fatma Suleiman, Ingrid Wolfe, Angela C. Flynn, Majella O'Keeffe and Lucilla Poston. Kathryn V. Dalrymple had overall responsibility for the manuscript.

## CONFLICTS OF INTEREST

The authors declare no conflicts of interest.

## Supporting information

Supporting information.

## Data Availability

Data sharing is not applicable to this article as no new data were created or analysed in this study.
