# Supplementary material for: Preventing and treating childhood overweight and obesity in children up to 5 years old: A systematic review by intervention setting
Source: Matern Child Nutr. 2022 Mar 25;18(3):e13354. doi: 10.1111/mcn.13354 (PMC9218326; doi:10.1111/mcn.13354)
Supplement: Supplementary file 1 — Supporting information. [file MCN-18-e13354-s001.docx]

Figure S1 – Study Quality

| **Study Author** | **Design** | **Randomisation** | **allocation concealment** | **participant drop-out** | **Overall risk of bias** |
| --- | --- | --- | --- | --- | --- |
| **Bellows et al. 2013** | RCT | Unclear | Unclear | High | High |
| **Berry et al. 2011** | RCT | Low | Unclear | Unclear | High |
| **Black et al., 2020** | RCT | Low | Low | High | Moderate |
| **Bocca et al., 2012** | RCT | Low | Unclear | Low | Moderate |
| **Campbell et al., 2013** | RCT | Low | Low | Low | Low |
| **Daniels et al., 2013** | RCT | Low | Low | High | Moderate |
| **De la Haye et al., 2019** | Pilot RCT | Unclear | Unclear | High | High |
| **Fisher et al., 2019** | RCT | Low | Low | Low | Low |
| **Fitzgibbon et al., 2011** | RCT | Unclear | High | Low | Moderate |
| **Haines et al., 2018** | Pilot RCT | Low | Unclear | Low | Low |
| **Helle et al., 2019** | RCT | Low | Low | High | Moderate |
| **Hodgkinson et al., 2019** | CRT | Low | Unclear | Low | Moderate |
| **Kim et al., 2019** | RCT | Unclear | Unclear | High | High |
| **Lumeng et al. 2017** | RCT | Unclear | Unclear | Low | High |
| **Martínez-Andrade et al., 2014** | CRT | Low | Low | High | Moderate |
| **Natale et al., 2014** | RCT | Unclear | Unclear | Low | High |
| **Natale et al., 2017** | RCT | Unclear | Unclear | Unclear | High |
| **Nystrom et al., 2017** | RCT | Low | Low | Low | Low |
| **Quattrin et al., 2014** | RCT | Low | Unclear | Low | Low |
| **Salazar et al., 2014** | RCT | Unclear | Unclear | Unclear | High |
| **Stark et al., 2011** | Pilot RCT | Low | Low | Low | Low |
| **Sherwood et al., 2015** | Pilot RCT | Unclear | Unclear | Low | Moderate |
| **Skouteris et al., 2016** | RCT | Low | Low | Low | Low |
| **Stookey et al., 2017** | CRT | Low | Low | Low | Low |
| **Tomayko et al., 2016** | RCT | Low | High | High | High |
| **Verbestel et al., 2014** | CRT | Unclear | Unclear | Low | Moderate |
| **Wall et al., 2019** | Double blind RCT | Low | Low | Low | Low |
| **Walton et al., 2016** | RCT | Low | Low | Low | Low |

**Supplementary information 2: Medline search strategy**

MEDLINE and EMBASE (1^st^ January 2000-31^st^ December 2020)

(P) Participant

1 exp child/

2 child*.mp.

3 small child*.mp.

4 early chidlhood.mp.

5 early years.mp.

6 school child*.mp.

7 exp infant/

8 infan*.mp.

9 toddler.mp.

10 exp animals/ not humans.sh.

(I) Intervention

11 exp diet/

12 intervention.mp.

13 counceling/ or counseling.mp.

14 education.mp

15 energy intake/ or dietary intake.mp.

16 nutrition* behavio?r.mp

17 health* eating.mp.

18 diet*.mp.

19 diet* intake.mp.

(O) Outcomes

20 obes*.mp.

21 exp obesity/

22 over?weight.mp.

23 weight change$.mp.

24 weight gain/

25 adiposity.mp.

26 body composition.mp.

27 (bmi or body mass index).mp.

(C) Combinations

28 #1 OR 2

29 #3-9 OR

30 #11-16 OR

31 #17-19 OR

32 #20-27 OR

33 #28-32 AND

34 #33 not 10

35 Limit #34 to yr="2000-current"
